# Co-Designing the MOSAIC mHealth App With Breast Cancer Survivors: User-Centered Design Approach

**DOI:** 10.2196/59426

**Published:** 2024-12-09

**Authors:** Betsey Zenk Nuseibeh, Shelley A Johns, Patrick C Shih, Gregory F Lewis, Tayler M Gowan, Evan J Jordan

**Affiliations:** 1 School of Public Health Indiana University Bloomington, IN United States; 2 School of Medicine Indiana University Indianapolis, IN United States; 3 Luddy School of Informatics, Computing, and Engineering Indiana University Bloomington, IN United States; 4 Center for Health Services Research Regenstrief Institute Indianapolis, IN United States

**Keywords:** breast cancer survivors, acceptance and commitment therapy, mHealth app, user-centered design, depression, anxiety, therapy, app, breast cancer, expert, designer, psychosocial, need, co-design, MOSAIC, mobile acceptance and commitment therapy stress intervention, interviews

## Abstract

**Background:**

Breast cancer is the world’s most prevalent cancer. Although the 5-year survival rate for breast cancer in the United States is 91%, the stress and uncertainty of survivorship can often lead to symptoms of depression and anxiety. With nearly half of breast cancer survivors living with stress and symptoms of depression and anxiety, there are a significant number of unmet supportive care needs. New and potentially scalable approaches to meeting these supportive care needs are warranted.

**Objective:**

This study aimed to engage breast cancer survivors and acceptance and commitment therapy (ACT) content experts in user-centered design (UCD) to develop a mobile health app (MOSAIC [Mobile Acceptance and Commitment Therapy Stress Intervention]) using stress intervention strategies.

**Methods:**

We held 5 UCD sessions with 5 breast cancer survivors, 3 ACT content experts, 2 user experience design experts, and 1 stress expert facilitator over the course of 10 weeks. The sessions were developed to lead the 10 co-designers through the 5-step UCD process (eg, problem identification, solution generation, convergence, prototyping, and debriefing and evaluation). Following the fifth session, a prototype was generated and evaluated by the 5 breast cancer survivors and 3 ACT experts using the System Usability Scale, Acceptability E-scale, and a brief set of semistructured interview questions.

**Results:**

The 10 co-designers were present for each of the 5 co-design sessions. Co-designers identified 5 design characteristics: simple entry with use reminders (behavioral nudges), a manageable number of intervention choices, highly visual content, skill-building exercises, and social support. A total of 4 features were also identified as critical to the use of the tool: an ACT and breast cancer–specific onboarding process, clean navigation tools, clear organization of the interventions, and once-per-week behavioral nudges. These requirements created the foundation for the app prototype. The 5 breast cancer survivors and 3 ACT co-designers evaluated the app prototype for 1 week, using an Android smartphone. They rated the app as usable (mean 79.29, SD 19.83) on the System Usability Scale (a priori mean cutoff score=68) and acceptable (mean 24.28, SD 2.77) on the Acceptability E-scale (a priori mean cutoff score=24).

**Conclusions:**

Through the UCD process, we created an ACT app prototype with 5 breast cancer survivors, 3 ACT experts, and 2 UCD designers. The next step in our research is to continue the assessment and refining of the prototype with additional breast cancer survivors. Future work will pilot-test the app to examine the feasibility of a large-scale, randomized control trial. Studies will enroll increasingly diverse breast cancer survivors to broaden the generalizability of findings.

## Introduction

### Background

Over 7.8 million women worldwide have been diagnosed and are living with breast cancer, making it the world’s most prevalent cancer [1,2]. Due to earlier detection and targeted treatment, the 5-year breast cancer survival rate is 91% in the United States [3]. After treatment, breast cancer survivors report high levels of stress [4] and clinically significant symptoms of depression and anxiety [5,6]. With a large number of breast cancer survivors experiencing symptoms of depression and anxiety, they have been shown to have unmet supportive care needs [7,8]. Access to in-person supportive care is often limited by clinician shortages, financial constraints, and time available to attend therapy sessions [9-13]. Mobile health (mHealth) apps may offer a scalable solution for breast cancer survivors motivated to learn adaptive coping skills to reduce stress and symptoms of depression and anxiety [8,14-16]

mHealth apps show promise in the treatment of the mental health of breast cancer survivors [15,17,18] and are particularly effective when created through user-centered design (UCD) [19-22]. This iterative process creates highly engaging and effective mHealth apps, requiring collaboration between end users, content experts, and user experience (UX) designers [23,24]. Clinical resources have not kept up with the quickly growing number of breast cancer survivors [25,26], and breast cancer survivors are open to technology-based support [27,28]. However, while recent reviews found that mHealth has an effect on decreasing stress for breast cancer survivors [17], there is a need for more evidence-based, psychological mHealth interventions with high-quality clinical trials.[25,29,30]

Acceptance and commitment therapy (ACT) is an empirically supported behavioral treatment for a diverse range of problems in living [31]. ACT has demonstrated efficacy in reducing symptoms of depression and anxiety in clinical and nonclinical populations [32]. Recent meta-analyses have also supported the use of ACT in adults with cancer in reducing stress, [33,34] and symptoms of depression and anxiety [33-36]. Furthermore, ACT is listed as 1 of several evidence-based treatments for adult cancer survivors with moderate symptoms of anxiety in the American Society of Clinical Oncology clinical practice guidelines for depression and anxiety [37]. ACT shows promise when used with breast cancer survivors experiencing stress and symptoms of depression and anxiety [33]. Finding skilled ACT therapists can be challenging [33]; thus, alternative delivery forms (eg, mHealth and telephone) for ACT have been explored by clinical psychologists working with patients with cancer [33,38,39]. While ACT-based mHealth interventions have shown feasibility with patients with metastatic breast cancer [40], an ACT-based mHealth app has not been developed to be used exclusively with breast cancer survivors.

### Objectives

The purpose of this study was to engage breast cancer survivors and ACT content experts in developing an mHealth app using stress management strategies. We conducted UCD with breast cancer survivors and a multidisciplinary team of experts in stress, UX design, ACT, cancer survivorship, and clinical psychology. In this paper, we describe the design session process with breast cancer survivors and the resulting app prototype. The process description will be useful to mHealth app designers intending to use the UCD approach. The incorporation of UCD into the mHealth app design process has the potential to fill the gap of unmet care needs for breast cancer survivors motivated to use mHealth to learn adaptive coping skills, through the creation of an app by the user for the user.

### Conceptual Framework

The use of mHealth apps for behavior change to manage stress and symptoms of depression and anxiety for breast cancer survivors has shown effectiveness [15,17,30,41]. However, many mHealth apps go unused because they fail to meet the needs of the patient, the clinician, or both [23]. The proposed solution, endorsed by the World Health Organization, [22] and International Standards Organization [42], is to use UCD, bringing the end user and content experts together with UX designers to co-design an mHealth app [23,43,44].

UCD in mHealth is an iterative, multidisciplinary process that actively includes the end user to identify their unmet needs and technology requirements, maximizing uptake of the final product [45,46]. The multidisciplinary team includes the end user, content experts, UX designers, and facilitators adept in leading the team toward the end goal. The iterative process begins with a needs assessment of both the end user and the content experts, including interviews and focus groups [19,23,44,47]. This initial step should also assess the context of use including the motivation of the app user, the user’s goals and strategies, activities, tasks, and complexities that can arise given the needs of the users [43]. As the sessions progress, the UX designers bring simple prototype sketches to the team of users and content experts for continued evaluation, refinement, and further iteration [19,21,24]. This process [48] typically includes five steps, which are (1) problem identification, (2) solution generation, (3) convergence, (4) prototyping, and (5) initial evaluation [49].

Our multidisciplinary team was comprised of breast cancer survivors (the end user), content experts, UX designers, and a stress expert facilitator. Based on our team’s previous work showing the feasibility and promise of ACT in reducing fear of recurrence and symptoms of anxiety and depression in breast cancer survivors [50]; clinical practice guidelines recommending ACT for the treatment of anxiety in cancer survivors [37]; and other evidence supporting ACT in reducing symptoms of anxiety, depression, and stress in breast cancer survivors [33,34], we chose ACT as the focus of the app we developed. We invited ACT content experts to guide stress intervention development with our end users. ACT is a behavioral psychotherapeutic approach designed to increase psychological flexibility in coping with difficult internal experiences (eg, thoughts, feelings, bodily, and sensations) [31,51,52] and is particularly relevant in a cancer context, where stress and symptoms of depression and anxiety are common [33,53-55]. In the context of ACT, psychological flexibility is defined as “contacting the present moment as a conscious human being, fully and without needless defense, and persisting with or changing a behavior in the service of chosen values” [31]. To encourage psychological flexibility, ACT interventions focus on three core pillars, which are (1) being centered in the present moment, (2) staying open to direct experience (eg, emotions), and (3) engaging in freely chosen values [56]. ACT as a therapeutic treatment modality for survivors has a growing evidence base [37,57-59], supporting survivors in cultivating present-moment awareness and disentangling from rigid thoughts about themselves and their cancer [60].

## Methods

### Study Design

Consistent with UCD principles, we incorporated the end user in every step of the app prototype development [19]. A total of 5 in-person UCD sessions were held biweekly from September 2022 to November 2022, with 10 co-designers present at each session. Sessions were audio recorded, each lasting 2 hours. The sessions were facilitated by the lead investigators, and 1 research team member was present to take field notes. A total of 2 UX designers joined for observation during the first 2 sessions and then began prototype design development in sessions 3 through 5. Information gathered from the co-designers in each session was used for prototype development in the 2 weeks between sessions, and feedback from the co-designers was requested the following session. The session content is outlined in Table 1.

**Table 1:** Design session number, design stage, and activities employed in each design session.

Session	Design stage	3-stage structure and activity
1	Problem identification	Priming: introduction to stage 1 of the MOSAIC^a^ study Design activity: name stressors related to survivorship; name stressors unrelated to survivorship; categorize according to frequency and intensity; and place on stress frequency and intensity matrix Debrief: summarize discussion
2	Solution generation	Priming: review the purpose of stage 1 of the MOSAIC study; review stressors identified in session 1 Design activity: ACT^b^ refresh, ACT exercise practice, and harvest preferred ACT exercises Debrief: summarize discussion
3	Convergence	Priming: review preferred ACT exercises named in session 2 Design activity: Gather input regarding when and where stressors occur, time spent thinking about stressor, preferred exercise, and format Debrief: summarize discussion
4	Prototyping	Priming: review ACT exercises identified as priorities for app inclusion in session 3 Design activity: provide samples for 5 key parts of the app, soliciting input on format and functionality Debrief: summarize discussion and evaluation of app
5	Debriefing and evaluation	Priming: prototype walk-through Design activity: “think-aloud” interaction with prototype Debrief: prepare for the SUS^c^

^a^MOSAIC: Mobile Acceptance and Commitment Therapy Stress Intervention.

^b^ACT: acceptance and commitment therapy.

^c^SUS: System Usability Scale.

### Participants and Recruitment

We recruited 5 breast cancer survivors from a cohort of breast cancer survivors participating in a randomized controlled trial evaluating behavioral interventions (including ACT) for clinical fear of cancer recurrence (R01CA255480) for the UCD sessions. We also recruited 3 ACT experts and 2 UX designers. A total of 10 co-designers are within the standard UCD research range [61] while reducing the potential for group thinking and ensuring ample time for providing perspective. Breast cancer survivors met study inclusion criteria if they (1) were ≥18 years of age, (2) were diagnosed with stage I to III breast cancer, (3) had completed breast cancer treatment ≤5 years previous (ongoing endocrine therapy was allowed), (4) had previously participated in an ACT intervention, and (5) were able to read and speak English. Breast cancer survivors were excluded from study participation if they had comorbidities that would impair participation in the study, including reduced cognitive function, severe depressive symptoms, active substance abuse, uncontrolled bipolar disorder, psychosis, or schizophrenia. Demographic data including gender, age, race, and ethnicity were collected*.*

### Procedures

The 5 sessions were developed to lead 5 breast cancer survivors, 3 ACT experts, and 2 UX designers in co-creating an ACT-based mHealth app to assist in managing stress. To maximize response input, UCD sessions included visual and aural questions. Co-designers engaged in both written and verbal discussion of topics. We used a 3-stage structure for each session: priming, a design activity, and debriefing, as introduced by Jolliff et al [62]. The activities used in each UCD session are presented in Table 1. Sessions were collaboratively planned by the research team including stress and ACT experts. Co-designers received a US $125 gift card at the completion of each session. Notably, all sessions were audio recorded and transcribed verbatim.

We briefly describe the 5-step design process below, followed by the results.

#### Design Session 1: Problem Identification

The goal of design session 1 was to identify stressors breast cancer survivors have felt during their survivorship. Co-designers were provided with sticky notes and asked to spend 10 minutes identifying experienced stressors from diagnosis to treatment to survivorship. These responses are reported in the *Results* section. We then asked survivor co-designers to spend 10 minutes identifying stressors experienced during survivorship not related to cancer (daily stressors). Each stressor was assigned a frequency rating from 1 to 4, which are (1) more than once per day, (2) about once per day, (3) several times per week, and (4) less than once per week, and an intensity rating from 1 to 4, which are (1) extremely stressful, (2) very stressful, (3) somewhat stressful, and (4) a little bit stressful. Survivor co-designers placed their stressors on 1 of 4 quadrants, which are (1) high intensity and high frequency, (2) high intensity and low frequency, (3) low intensity and high frequency, and (4) low intensity and low frequency. Breast cancer survivors were asked to identify missing stressors if any stressors should be moved to a different group and which group of stressors should be focused upon during the next step. After the session, we collected the frequency and intensity chart to analyze stressor themes in preparation for session 2.

#### Design Session 2: Solution Generation

The goal of session 2 was to generate ACT exercises (ACTivities) to address high-frequency and high-intensity stressors experienced by survivor co-designers to be included in the app. After reviewing the three ACT core pillars, which are (1) being centered in the here and now, (2) staying open to direct experience (eg, emotions), and (3) engaging in freely chosen values [56], breast cancer survivors were asked to share memorable ACTivities they found useful when experiencing past stressors and ACTivities presently useful.

#### Design Session 3: Convergence

The goal of session 3 was to identify survivor co-designer’s perception of when and where stressors occur, and the amount of time spent coping with specific stressors. After the generation of these responses, breast cancer survivors were asked to suggest ACTivities that might be helpful in addressing the stressors and the possible format (eg, audio, video, and worksheet) of the exercise.

#### Design Session 4: Prototyping

Before session 4, the UX designers created multiple prototypes of mHealth app elements to discuss with the UCD team. The co-design team discussed the introduction and onboarding page, the home page and navigation tools, the organization of the ACTivities, the format of the ACTivity pages, and the format of a weekly check-in. To facilitate discussion, examples were presented and discussed in terms of features and functionality.

#### Design Session 5: Initial Evaluation

Before session 5, the feedback from session 4 was incorporated into the app content and design by the UX designers. The goal of session 5 was to lead the breast cancer survivors through an app prototype to explore the usability of content and design. This session involved breast cancer survivors and content experts interacting with the prototype app, providing input on the interface, functions, and overall look and feel. A key step in UCD is to provide prototypes to a small number of intended users to try real-world tasks and scenarios [63].

### Measures

The UX designers incorporated feedback from the end users (breast cancer survivors) and ACT experts into the prototype design after session 5. The app was coded and a full, working prototype was created for the Android platform. Each co-designer, including the 5 breast cancer survivors and the 3 ACT content experts, received an Android smartphone preloaded with the app in the mail. We asked them to each use the app for a week and then complete the System Usability Scale (SUS); the Acceptability E-scale; and additional written, short answer probes regarding their experience with the prototype.

#### SUS and Acceptability E-scale

The 10-item SUS is a validated questionnaire containing ten 5-point items with alternating positive and negative tone, rated from strongly disagree (1) to strongly agree (5) [64,65]. The alternating statement tone requires score conversion [65]. Adjusted SUS scores range from 0 to 100, with higher scores indicating higher usability [65,66]. Sample statements include “I thought MOSAIC was easy to use” and “I thought there was too much inconsistency in MOSAIC.” Based on recommendations from the validated SUS for mHealth apps, we set a priori cutoff scores for a passable app at 68. Apps with SUS scores at this level or higher are considered usable and likely to experience user uptake [67].

The Acceptability E-scale is a questionnaire validated in adults with cancer that contains 6 questions assessing the acceptability of technology-based products, each with 5 response options [68]. A response of 1 indicates a negative response, 3 indicates a neutral response, and 5 indicates a positive response. Sample questions include asking about the ease and enjoyability of the product and the amount of time required to complete product tasks. Scores range from 6 to 30. A score of 24, or 80% of the highest possible summary score, is the recommended cutoff for adequate acceptability of a technology-based product as rated by the participants [68]. Thus, we set our a priori cutoff score at 24 for product acceptability [68].

#### Data Analysis

Design session data were analyzed using inductive thematic analysis [69]. The audio recordings from each co-design session were transcribed and analyzed by a team of 2 coders (BZN and TMG). Initial coding was inductive, with new codes emerging from responses without a priori specification [69]. Responses were independently coded and then discussed to refine and establish consensus. Once responses were thematically coded, they were deductively organized according to the co-design session plan (Table 1), including human factor elements (eg, design, content, and ease of use) [70]. Themes were brought to 2 additional team members (SAJ and EJJ) for further refinement through consensus discussion. Ongoing results were used to recommend design requirements, prototype alterations, and the agenda for the next co-design session. This process occurred after each co-design session. Following the fifth co-design session, final categorical codes were placed into a table, and individual response codes were analyzed for consistency and saliency within categorical codes. Relevant quotes for each of the themes were summarized and used in this manuscript.

Descriptive statistics were used to analyze demographic data. Initial usability and acceptability were quantitatively measured with the SUS and Acceptability E-scale, which were scored according to requirements [66,68].

### Ethical Considerations

This study was evaluated and approved by the Indiana University Institutional Review Board (#15829). Qualitative study data was deidentified to protect confidentiality. All participants provided informed consent through an information sheet. The participants received US $675 as compensation for their contribution to the 5 co-design sessions and their final evaluation of the app prototype.

## Results

### Demographics

A total of 5 co-design sessions took place in person between September and November 2022. The group contained 5 breast cancer survivor co-designers and 3 ACT content experts. ACT experts had practiced a mean of 10.3 (SD 7.1) years*.* All co-designers were living in Indiana. The demographic characteristics of the sample are presented in Table 2.

**Table 2:** Co-design session participant demographics (n=8).

Demographic characteristics			Breast cancer survivor co-designers (n=5)		ACT^a^ content experts (n=3)
**Age (years), mean (SD)**			54 (3.4)		41 (15.2)
**Gender, n (%)**					
	Female	5 (100)		2 (67)	
	Male	0 (0)		1 (33)	
**Race and ethnicity, n (%)**					
	White	4 (80)		3 (100)	
	Black or African American	1 (20)		0 (0)	

^a^ACT: acceptance and commitment therapy.

### SUS and Acceptability E-scale

After session 5, the mean SUS score of the MOSAIC (Mobile Acceptance and Commitment Therapy Stress Intervention) app prototype was 79.29 (SD 19.83), indicating that both breast cancer survivors and ACT clinicians found the product acceptable with good to excellent potential for use [66]. The summed total of mean Acceptability E-scores was 24.28, indicating the initial acceptability of the prototype. Both the SUS and Acceptability E-scores were at or above the cutoff points set a priori for app usability and acceptability. The mean and SD of each Acceptability E-score as well as the total are presented in Table 3.

**Table 3:** Acceptability E-scores for MOSAIC^a^ following co-design session 5.

Statement	Score, mean (SD)
How easy was MOSAIC for you to use?	4.43 (0.79)
How understandable were the questions in MOSAIC?	4.14 (1.07)
How much did you enjoy using MOSAIC?	4.00 (0.82)
How helpful was MOSAIC in addressing your stressful experience?	4.00 (0.82)
Was the amount of time it took to complete an exercise in MOSAIC acceptable?	3.71 (1.89)
How would you rate your overall satisfaction with MOSAIC?	4.00 (1.00)
Total	24.28 (2.77)

^a^MOSAIC: Mobile Acceptance and Commitment Therapy Stress Intervention.

### Qualitative Results

Qualitative input from the 8 co-designers was analyzed using inductive thematic analysis by a team of 2 coders and categorized into 3 main domains: mHealth app content, design characteristics, and features and functions.

#### mHealth App Content

Guided by the iterative process of UCD, our multidisciplinary team including breast cancer survivors (the end user), ACT content experts (the content experts), UX designers, and facilitators identified three stress-based themes unique to breast cancer survivors: (1) self (eg, fear of recurrence), (2) relationships (eg, impact of cancer on children or other loved ones), and (3) work and financial (eg, financial stability), as outlined in Table 4. These themes were linked to breast cancer survivors’ preferred ACT interventions and reviewed with breast cancer survivors by the ACT content experts to begin prototyping self-care ACTivities through the app.

**Table 4:** Stressors identified as unique to breast cancer survivorship by co-designers in Session 1.

Stress-based theme	Subthemes	Example quotations
Self	Fear of cancer recurrence, pain, doctor visits, change in appearance, and cognitive burden of decision-making	Once you’re done with treatment, you’re supposed to have this big party…and that was the worst. Because at least when I was in treatment, I was doing something and I was being active. And once I was done, I really felt like lost for quite some time. I feel like that is another big hole. [SID 003] ...it is just the cognitive burden of all these decisions…every time I am like, “Did I make the right decision?” [Be]cause every single decision has pros and cons… [SID 004]
Relationships	Impact of cancer on children, impact of cancer on partner, and caregiving for aging parents	...being a caregiver is stressful and it can make you sick if you don’t take care of yourself in addition to taking care of the other person. [SID 001] ...my kids were four, six, and eight when I was diagnosed…they were really young. I had a lot of just fears for my future and what that would mean for them. [SID 003]
Work and financial	High expectations with limited internal and external resources, and the need for financial stability	I’m back to work, I have a pretty demanding job…Everyone knows I had cancer and I want to show them that I’m good, I can push forward. [SID 004] One thing that my husband and I have been talking about...was all the insurance is under me now, and if I get sick again and can’t continue working, then the whole family loses insurance. [SID 002]

#### Design Characteristics

Through content analysis of transcribed sessions, we identified 5 design characteristics: simple entry with the use of reminders (behavioral nudges), a manageable number of intervention choices, highly visual content, skill-building ACTivities, and social support. The design characteristic themes are summarized in Table 5.

**Table 5:** App design characteristics and features and functions identified by breast cancer survivors as critical during app use.

Theme and subtheme			Exemplary quote
**Design characteristic**			
	Simple entry with use reminders	*initial question each time like “how do you feel today?” to help direct to the next level of the app.* [SID 002]	
	A manageable number of intervention choices	*limited choices, that way you’re not overwhelmed and you’re not like ‘well, I don’t know which one to pick.* [SID 006]	
	Highly visual content	*I think any way you can help people visualize what they need to do, especially on an app, that’s gonna be super helpful*. [SID 004]	
	Include in-the-moment and skill-building activities	*If you’re talking about someone who can’t get into to see a therapist for 2 months, it would be great to have the quick fix, for lack of a better word. But...where I think the true healing comes, is the skills that you start practicing regularly.* [SID 003]	
	Social support	*Remember, other people love and want to help. They just don’t know how.* [SID 003]	
**Features and Functions**			
	Acceptance and commitment therapy and breast cancer–specific onboarding process	*critical to acknowledge that breast cancer is why you’re even in this app...that’s where some of the stress comes in. We know we have it* [cancer]*. We know we’ve been through this, but we really don’t acknowledge that we actually had it. We continue to hold ourselves to standards, regardless of the trials and tribulations of cancer and treatment*. [SID 001]	
	Clean home page and navigation tools	*I like using three* [offered choices]. *And I think the language is* [currently] *inaccessible to* [some who may be] *using it, so we might think about simplifying it. But I like the idea of three.* [SID 004]	
	Clear intervention organization	*It’s just a very simple thing. Yes, it was useful. I’m done, I can move on and get back to whatever I need to do. And then it comes up again later. I really liked that. Let me try that again*. [SID 002]	
	Behavioral nudges once per week	*When I was in the middle of chemo, once a week, maybe even more frequently was important. But now, five years out, I don’t need a weekly check-in anymore*. [SID 002]	

### Simple Entry with Use Reminders (Behavioral Nudges)

There was a stated need for framing the intent upon opening the app. One breast cancer survivor noted a preference for a short and consistent message every time the app opens to “frame the person’s mind before they get into anything” (SID 003).

Another breast cancer survivor said that it could be an “initial question each time like ‘how do you feel today?’ to help direct to the next level of the app” (SID 002).

Another breast cancer survivor suggested that upon entry the app might assess the user’s emotions, explaining that she prefers to “throw out my feelings before I can organize them” (SID 003).

### A manageable Number of Intervention Choices

Breast cancer survivors discussed the necessity for autonomy when choosing activities while not being overwhelmed with too many possibilities:

I like that there’s choices, too. [Be]cause...[SID 001] might like one sheet and I might use it differently. So, it’s nice to have options.

SID 003

All people learn differently, some by reading, some by seeing, some by acting it out.

SID 005

**Figure 1:**
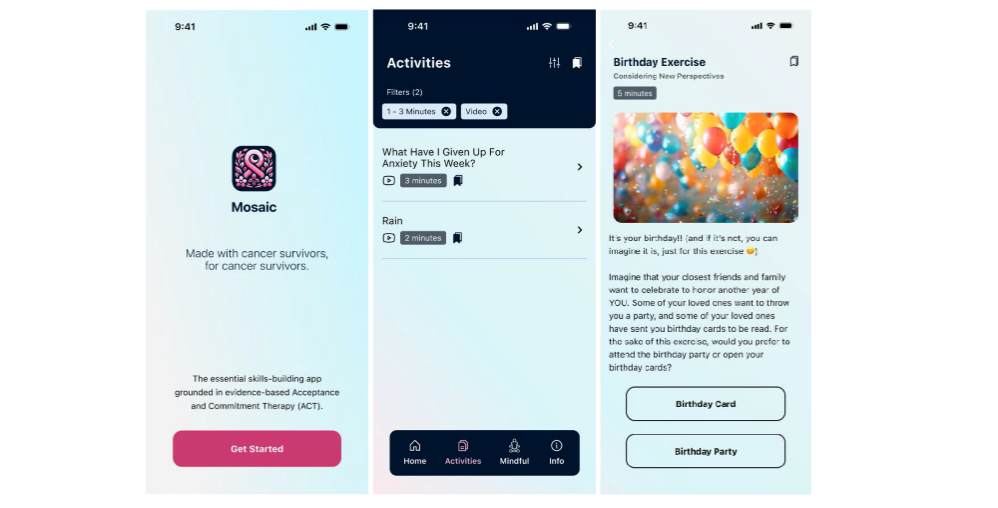
Examples of MOSAIC (Mobile Acceptance and Commitment Therapy Stress Intervention) app screens: (A) launch screen, (B) home page, and (C) values commitment page.

Furthermore, breast cancer survivors noted that the act of receiving a cancer diagnosis felt like taking away power, and the provision of choice through the app would be a directional step back toward that power (Figure 1). While recognizing the need for choices, they also discussed the importance of having “limited choices, that way you’re not overwhelmed and you’re not like ‘well, I don’t know which one to pick’” (SID 006).

### Highly Visual

Frequently discussed by breast cancer survivors was the need for highly engaging, visual content. Breast cancer survivors stated that visualizing the exercises really gives them the ability to notice and feel the experience in their body. Particularly when interacting with an app through self-management of anxiety, breast cancer survivors noted that the inclusion of colors and other visual elements could produce higher levels of activity and engagement.

You need to visualize it [feeling or thought] I’m a visual person, so I think you need to...actually see it. I think that’s helpful.

SID 001

I think any way you can help people visualize what they need to do, especially on an app, that’s gonna be super helpful.

SID 004

I wonder if there’s something where you have an icon or something that you move yourself. It’d be visual and active.

SID 002

### Include in the Moment and Skill Building ACTivities

Breast cancer survivors agreed that many times they would be using the app to assist with anxiety in the moment it occurs, saying:

Anxiety might hit you in different contexts, so if you’re sitting in the doctor’s office you might need one thing. If you’re trying to go to sleep and you’ve been up for two hours, you might need something else.

SID 004

However, they also suggested adding skill-building ACTivities:

Maybe you could click to get more information [on a meaningful intervention]. Maybe there’s something like “this is an acute issue versus I’m building my skills,” and that would take you down a different path.

SID 002

One breast cancer survivor noted the absolute necessity for both:

If you’re talking about someone who can’t get in to see a therapist for two months, it would be great to have the quick fix, for lack of a better word. But...where I think the true healing comes, is the skills that you start practicing regularly.

SID 003

### Social Support

Throughout the discussions, breast cancer survivors shared their desire for social support. Breast cancer survivors needed to let others know that they were not alone and that what they had gone through could benefit someone else:

I felt just like you. I did this...it’s all just trying to figure out what have other people experienced and what’s worked for them and what hasn’t.

SID 004

People say what they did and then you’re like, oh, I’ll try that!

SID 005

They also recognized the acute loneliness that comes with treatment and survivorship, and asked for comforting reminders to be strategically placed in the app:

Remember, other people love and want to help. They just don’t know how.

SID 003

#### Features and Functions

Using content analysis, we identified elements within the features and functions of the mHealth app that were critical to breast cancer survivors during use. We probed co-design participants for information regarding onboarding, home page and navigation, intervention organization, and behavioral nudges. The features and functions themes are summarized in Table 5.

### Onboarding

Upon opening the mHealth app, breast cancer survivors stated that they wanted to see and hear the words “breast cancer” immediately (Figure 1). They wanted an identification of why they had arrived (stress), saying it is “critical to acknowledge that breast cancer is why you’re even in this app...that’s where some of the stress comes in. We know we have it [cancer]. We know we’ve been through this, but we really don’t acknowledge that we actually had it. We continue to hold ourselves to standards, regardless of the trials and tribulations of cancer and treatment” (SID 001).

A primary step in the ACT process is the identification of the participant’s values. Both breast cancer survivors and ACT content experts felt value identification was a necessary part of the user’s app initiation (Figure 1). One breast cancer survivor emphasized that “All the rest of the work anchors in the values” (SID 002).

They were initially presented with a horizontal list of values and discussed the desire instead for a vertical list with the “option to type in a value that’s not on the list” (SID 003).

A discussion of values accountability, stress, and the ACT process showed a preference for a slider rather than definitive questions to avoid the feeling of judgment and self-criticism. They stated the desire for “accounting for how breast cancer has affected your ability to live your values, [rather than] are you living into your values or not?” (SID 004).

### Home Page and Navigation

The discussion regarding navigation to interventions within the app identified the need for users to be able to choose an ACTivity-based on one of the following: (1) time, (2) current feeling, (3) core ACT pillar, or (4) type of stressor. While some discussed filters to aid navigation, others expressed distaste for filter navigation.

I’d rather be able to scroll through and pick it [an ACTivity] right there in front of me rather than having to click the little filter...I do not like filters.

SID 003

They acknowledged that the ability to choose or the need for a suggested ACTivity was dependent on the cognitive load induced by the stressor. Discussion arose regarding the number of choices provided, and the language in which choices are presented:

I like using three [offered choices]. And I think the language is [currently] inaccessible to (some who may be) using it, so we might think about simplifying it. But I like the idea of three.

SID 004

Regarding navigation of elements with many choices, such as values selection, the UX designers initially provided the option of both horizontal and vertical scrolling. This was vetoed by the group:

You should not have to scroll to the side and also down.

SID 002

### Intervention Organization

The organization of the intervention was introduced by the UX designers as a notification (“how are you feeling right now?”), followed by initiation of the intervention itself, and completed with an evaluative question like “Was this useful?”

**Figure 2:**
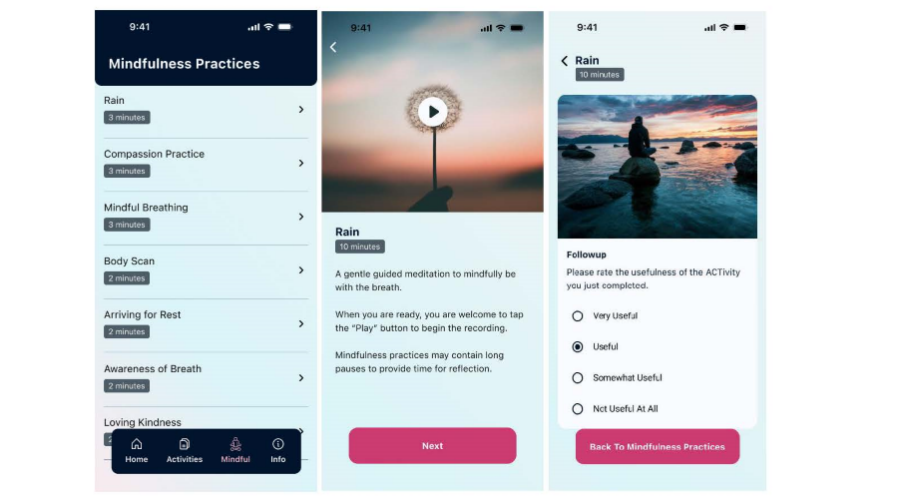
Examples of MOSAIC (Mobile Acceptance and Commitment Therapy Stress Intervention) app screens: (A) sample page of mindfulness ACTivity choices, (B) specific mindfulness ACTivity examples, and (C) evaluation of mindful ACTivities. ACTivity: acceptance and commitment therapy exercise.

Breast cancer survivors discussed at length the type of intervention that might pop up initially and ultimately recommended that there should be a choice, framed by the core pillars (Figure 2), stating “I do think you need some context [as] you become familiar” (SID 004).

Following participation in an ACTivity for stress reduction, both the breast cancer survivors and the ACT content experts determined that asking users if it was useful was critical. If they found the intervention useful, it would display later on the home page as a favorite (Figure 2):

It’s just a very simple thing. Yes, it was useful. I’m done, I can move on and get back to whatever I need to do. And then it comes up again later. I really liked that. Let me try that again.

SID 002

Breast cancer survivors also noted that after every intervention they felt it was important to remind users that they are not alone in their stress responses related to surviving cancer.

We want to remind them, what you are going through, you are not alone. It’s a continuous feeling that nobody else has gone through what you are feeling and we want them to remember, you are not alone.

SID 001

### Behavioral Nudges

Breast cancer survivors discussed digital behavioral reminders (nudges) within the app, recommending to the UX designers a standard set of nudges occurring once per week, with the option to check more or less depending on their place in survivorship. Some acknowledge that during treatment more check-ins may be useful, while farther into survivorship, intermittent check-ins may be adequate:

When I was in the middle of chemo, once a week, maybe even more frequently was important. But now, five years out, I don’t need a weekly check-in anymore.

SID 002

Breast cancer survivors were also concerned that, in keeping with ACT pillar 2, staying open to direct experience, nudges are not paired with pressure to change or any content that might elicit thoughts like “I’m broken, I need to fix myself” (SID 004).

Rather, breast cancer survivors preferred that nudges remain consistent with the language of ACT, filled with “...the kindness. The invitation. So, the invitation first, but then also the kindness. The nonjudgmental attitude” (SID 002).

## Discussion

### Overview

We used a UCD process to engage breast cancer survivors, ACT content experts, and UX designers in the design of a mHealth app using stress intervention strategies. The problem identification and solution generation exercises conducted during the first and second sessions of the co-design process identified stress-based themes and corresponding ACTivities salient to breast cancer survivors and ACT content experts, increasing the potential for technology uptake [19-22]. After identifying these elements, the breast cancer survivors, ACT content experts, and UX designers worked together to iterate and revise the prototype during sessions 3 through 5.

### Principal Findings

This study illustrates the potential for integration of a behavioral psychotherapeutic approach (ACT) with UCD to address a gap in supportive care for breast cancer survivors. Breast cancer survivors in this study recognized the current limitations of in-person psychosocial support [9,71], particularly a shortage of mental health clinicians trained to meet the unique needs of cancer survivors [11,72,73]. Throughout their discussions, they noted that the app could assist with stress and symptoms of depression and anxiety in the moment it occurs (eg, waiting for an appointment, experiencing sleeplessness), while also assisting the user in “true healing” (SID 003) through regular practice. Breast cancer survivors also highlighted the need for social supports to be woven throughout the app, even simply verification of belonging through recognition that the app was created by breast cancer survivors for breast cancer survivors.

In line with recent mHealth and cancer research [74], we incorporated the collaborative viewpoints of the end user and ACT and UX experts to encourage the use and uptake of our end product. We predetermined the use of ACT as the foundation for the design of the MOSAIC app because it has shown effectiveness in improving health outcomes for breast cancer survivors [33] and is a recommended evidence-based treatment for cancer survivors by the American Society of Clinical Oncology [37]. The 5 breast cancer survivor co-designers had each completed a previous ACT intervention program, and we also included 3 ACT content experts to complete the UCD process. Through their discussions, it was clear that the group of 8 co-designers in this study was invested in ensuring that the language throughout the app remained consistent with ACT core pillars while also being accessible to the user with less ACT experience. They noted that any language prompting app use (eg, behavioral nudges) should remain invitational and nonjudgmental [31]. The breast cancer survivors and ACT and UX experts included in the app development each maintained the integrity of their lived experience while contributing to the design, features, and functions of the prototype.

Following revisions and coding after the fifth session, we asked the 8 co-designers to use the app prototype on an Android smartphone for 1 week, evaluating their experience through the SUS, Acceptability E-scale, and short interview questions. Co-designers quantitatively evaluated the app as usable and acceptable. Participant responses to the short interview questions noted approval of the prototype while acknowledging functional errors requiring revision. Based on scores and short answer feedback, we will continue to iterate and revise the prototype in future evaluation stages.

### Comparison With Previous Work

Regarding the design characteristics of the prototype app, a number of design themes identified by breast cancer survivors and ACT content experts were consistent with the literature outlining stakeholder experience in UCD. For example, our results underscore the need for a variety of design facets, as users vary in their ACTivity preferences. This is highlighted in previous ACT-based mHealth app development discussing a need for both a variety of therapeutic modalities (eg, worksheet and video) and tools for interaction (eg, slider and lists) [75]. However, breast cancer survivors also discussed their need for limited choice to reduce cognitive burden. This paradox of variety, but not an overwhelming amount, is consistent with user evaluations of ACT mHealth apps [76]. A design theme that arose multiple times was the use of choice to promote a sense of autonomy. Our results highlight the need for a tool providing stress relief through intervention while further work is embedded with choice, bringing a sense of autonomy back to the user. This is consistent with the mHealth evaluation literature stating users are more motivated to engage with apps and products encouraging autonomy and emphasizing user choice [77].

Our results highlighted the need for multiple context-of-use options (eg, voice recordings, videos, and worksheets). This echoes previous research discussing the need for app use arising in a variety of environments (eg, doctor’s office and bedroom) with varying time available to engage with the exercises [76]. Our results also shone a light on the potential for social support in the app. Breast cancer survivor co-designers discussed a desire to provide testimonials and to inform future users they are not alone in their feelings. This is consistent with features in other ACT-based apps providing text from other users for instruction and motivation.[76]

Regarding features and functions, breast cancer survivors desire organizations to allow them to earmark activities that are effective for them, enabling them to return as needed. This is consistent with literature discussing “automated tailoring,” an effective and often requested feature in mHealth apps [77]. Breast cancer survivors discussed a desire for an appropriate number of reminders to engage (behavioral nudges) with the app. For this group, once per week was the recommended number. An external trigger such as a behavioral nudge is useful when used with the frequency needed for the population [77]. Behavioral reminders should remain as invitations to maintain ACT consistency while encouraging agency in-app use.

### Limitations

Our design group had a typical number of co-design participants for the UCD process while reducing the potential for group thinking and ensuring ample time for providing perspective. Multiple design sessions with the same co-design participants provided us with rich data for developing the initial prototype, and the method is supported by other researchers [78]. Because the group was small, we were able to find a mutually agreeable time to meet in person. However, the requirement of 5 in-person meetings may not have allowed for the inclusion of breast cancer survivors who are highly stressed, who may not have been able to participate in 10 total hours of research. In addition, current levels of perceived stress and symptoms of depression and anxiety were not evaluated as inclusion criteria, thus it is impossible to know the potential current levels of breast cancer survivors’ stress and symptoms of depression and anxiety. In short, the small number of co-designers did not allow for full representation of all potential users. Future evaluation of the app by breast cancer survivors will need to include a diverse sample of participants to ensure iterative feedback represents the spectrum of potential users.

The final MOSAIC app prototype coding was completed 6 months after the fifth design session. We maintained engagement with the co-designers during the app-building process. It is possible, however, that the length of time between the fifth design session and the final prototype evaluation by the co-design team was affected by the time between. In addition, the final prototype has a limited number of activities. The number was sufficient for our initial group of co-designers but may need to be increased to maintain engagement for longer evaluation periods [79]. Future work should increase the number of activity choices and content for breast cancer survivors, while continuing to test usability and feasibility.

While we felt it necessary for co-designers to be familiar with ACT and incorporate the completion of an ACT-based intervention as an eligibility criterion, we also recognize familiarity may be a limitation of the study. Current co-design participants are familiar with the intervention elements and ACT process, thus more explanation may be necessary for future users who are less familiar. mHealth apps can provide in vivo ACT training when it is most needed, for a variety of skill levels [79]. Therefore, future research should test the app with breast cancer survivors naïve to the ACT intervention process.

### Conclusions and Future Research

We developed a mHealth app using the co-design methodology, incorporating the preferences of both breast cancer survivors and ACT content experts. Our study contributes to the growing literature detailing the inclusion of the end users in mHealth design and evaluation. The next step of this research is to assess and refine the system usability of the app with 15 additional breast cancer survivors. We will then pilot-test the app to examine the feasibility of a larger-scale randomized controlled trial. Future studies will enroll increasingly diverse samples to broaden the generalizability of findings. Should studies show larger-scale feasibility and generalizability to diverse samples of breast cancer survivors, we believe the possible avenues for scalability include partnering with pharmaceutical or insurance companies to provide services closing the gap in unmet supportive care needs for breast cancer survivors.

## Abbreviations

ACT: acceptance and commitment therapy
ACTivity: acceptance and commitment therapy exercise
mHealth: mobile health
MOSAIC: Mobile Acceptance and Commitment Therapy Stress Intervention
SUS: System Usability Scale
UCD: user-centered design
UX: user experience

## Data Availability

The datasets generated and analyzed during this study are not publicly available but are available from the corresponding author on reasonable request.
